# Gut microbiota-derived extracellular vesicles exhibit diurnal regulation and activate hepatic gluconeogenesis

**DOI:** 10.1016/j.molmet.2025.102180

**Published:** 2025-06-06

**Authors:** Jian Tan, Jemma Justine Taitz, Duan Ni, Camille Potier-Villette, Gabriela Pinget, Tamara Pulpitel, Dragana Stanley, Ralph Nanan, Laurence Macia

**Affiliations:** 1 Charles Perkins Centre, The University of Sydney, Sydney, NSW, Australia; 2 School of Medical Sciences, Faculty of Medicine and Health, the University of Sydney, Sydney, NSW, Australia; 3 Sydney Medical School Nepean, the University of Sydney, Sydney, NSW, Australia; 4 Nepean Hospital, Nepean Blue Mountains Local Health District, Sydney, New South Wales, Australia; 5 School of Life and Environmental Sciences, Faculty of Medicine and Health, The University of Sydney, Sydney, NSW, Australia; 6 School of Health, Medical and Applied Science, Central Queensland University, Rockhampton, QLD, Australia

**Keywords:** Gut microbiota, Extracellular vesicles, Circadian rhythm, Gluconeogenesis, Glucose homeostasis, Metabolism

## Abstract

The circadian clock regulates tissue-specific homeostasis, and its disruption is associated with metabolic disorders. Both host metabolic processes and the gut microbiota exhibit diurnal regulation, and both contribute to the maintenance of glucose homeostasis (Thaiss et al., 2014; Bishehsari et al., 2020; Frazier et al., 2023) [1–3]. However, how the gut microbiota and the circadian rhythm interplay to control host glucose homeostasis is not fully understood. Here, we identified gut microbiota-derived extracellular vesicles (MEV) as a potential peripheral Zeitgeber (time cue) for the hepatic circadian clock, controlling hepatic gluconeogenesis. Host feeding patterns influence the gut microbiota, driving the diurnal production of MEV. Gut MEV levels coincide with the activity of hepatic gluconeogenesis, with overnight fasting associated with increased production of MEV by gut bacteria. MEV directly activates hepatic gluconeogenesis and chronic increase in MEV exposure impairs glucose homeostasis *in vivo*. Our finding highlights a mechanism by which the gut microbiota has co-evolved with the host to support its glucose needs during periods of energy demands (such as during fasting or starvation). On the contrary, an abnormal increase in MEV production, leading to dysregulated gluconeogenesis, may underlie various glucose-associated disorders, such as type 2 or gestational diabetes. Together, our data reconcile the gut microbiota and circadian rhythm in the control of host glucose homeostasis and metabolic health.

## Highlights

• Gut microbiota extracellular vesicle production is regulated by host feeding status.
• Gut microbiota extracellular vesicle activates hepatic gluconeogenesis.
• Gut microbiota extracellular vesicle influence the hepatic circadian clock.

## 1 Introduction

Glucose is a major energy source critical for the optimal function and survival of most cellular organisms. However, the availability of glucose fluctuates constantly, influenced by factors like nutrient availability and the organism's feeding state. Gluconeogenesis is a critical metabolic process that evolved to maintain euglycemia during periods of low glucose availability. In mammals, it occurs predominantly in the liver and is activated in response to low blood glucose levels, such as during fasting. This process is hormonally regulated and is primarily stimulated by cortisol and glucagon produced by the adrenal glands and the pancreas, respectively. The secretion of these hormones is also regulated by circadian rhythm [1], anticipatory to the rhythmic transition between feeding and fasting states. Indeed, gluconeogenic genes like phosphoenolpyruvate carboxykinase (PEPCK) and glucose-6-phosphatase (G6P) exhibit diurnal regulation, aligning with the activity of the master transcription factors CLOCK and BMAL1 [2], which govern the mammalian circadian clock. Recent evidence has highlighted a role for the gut microbiota in regulating host metabolism and metabolic health [3]. Notably, the absence of a gut microbiota led to lower blood glucose levels under both fed and fasted states [4], while microbial-derived metabolites like butyrate can directly stimulate hepatic gluconeogenesis [5]. Dysregulation of blood glucose levels is associated with metabolic disorders like type 2 diabetes [6] and gestational diabetes [7]. Modern lifestyle factors are linked to detrimental shifts in gut microbiota composition and their function [8,9]. This underscores the importance of understanding how gut microbes can influence host glucose homeostasis, and ultimately, metabolic health. Furthermore, little is known about how the gut microbiota regulates peripheral clocks, given the well-established consequence of circadian rhythm disruption to the development of metabolic disorders [[10], [11], [12]]. Current identified microbial factors that influence gluconeogenesis act indirectly by optimising intracellular hepatic metabolism [4], or directly by acting as substrates for gluconeogenesis [5]. While many of these pathways are rhythmically regulated due to host dietary behaviours, they do not directly affect circadian rhythm per se, with no reported effects on the mammalian clock system. Emerging evidence indicates that gut microbiota-derived extracellular vesicles (MEVs) play a significant role in the host-gut microbiota axis [[13], [14], [15]]. MEV are nanosized membrane structures produced primarily from the budding of the plasma membrane and can carry a wide range of products including DNA, protein, and lipids. There are evidence that bacterial-derived extracellular vesicles (EV) can influence host metabolic outcomes. For example, orally administered *Pseudomonas panacis*-derived EVs induce insulin resistance and impairs glucose metabolism in skeletal muscles [16] while *Akkermansia muciniphila* EVs improved the metabolic profile of high-fat induced diabetic mice [17]. It is unclear what role EVs produced by resident gut microbiota play on host gluconeogenesis and hence glucose homeostasis.

## 2 Results

### 2.1 Gut microbiota-derived extracellular vesicles directly activate hepatic gluconeogenesis

**Figure 1:**
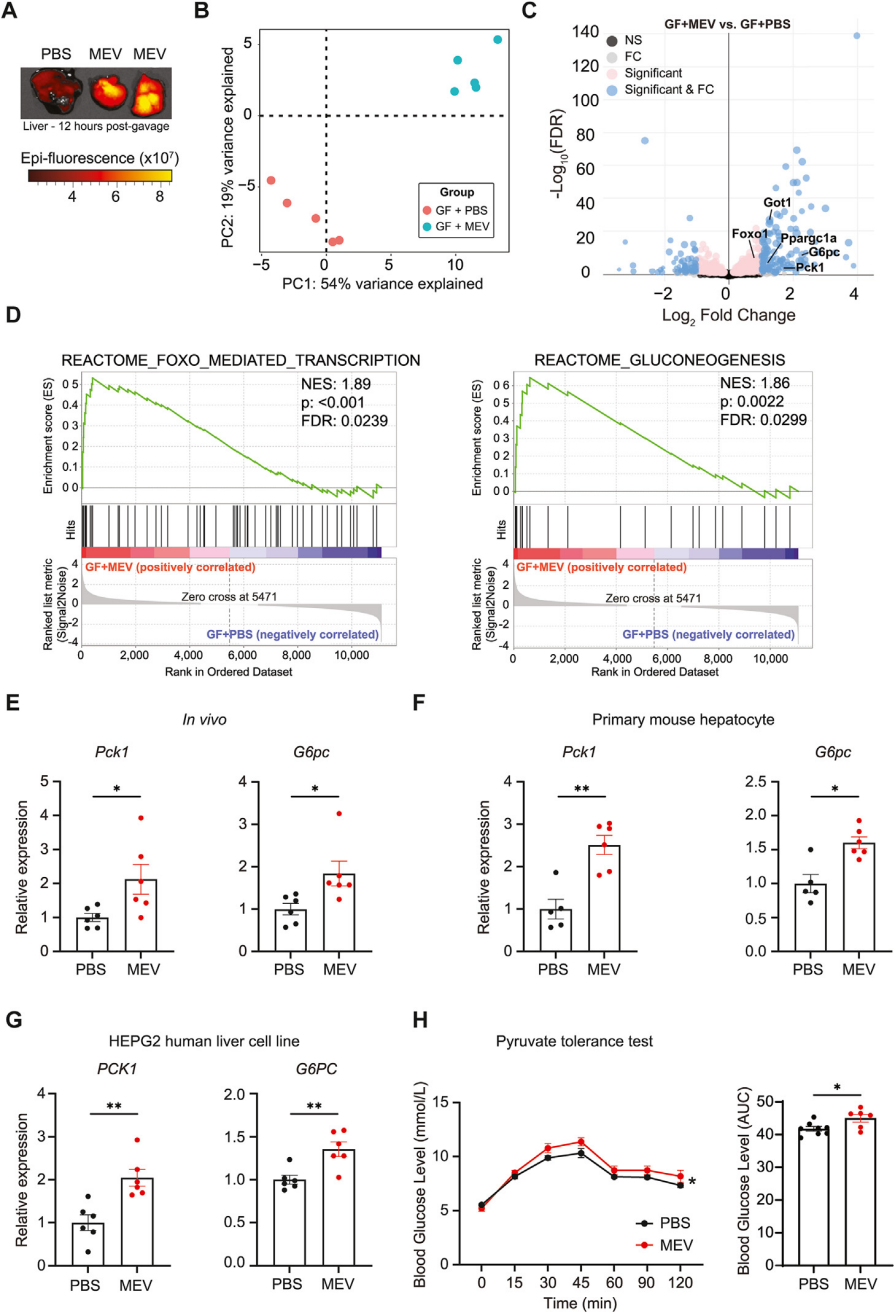
Gut microbiota-derived extracellular vesicles induce hepatic gluconeogenesis *in vivo* and *in vitro*. **(a)** Germ-free animals were orally administered 80 μg of DiD-labelled microbiota-derived extracellular vesicles (MEV) (n = 2) or PBS as control (n = 1). 12 h later, liver tissue was collected and fluorescence quantified *ex vivo* using the IVIS (In vivo imaging system). Fluorescence represented as Epi-fluorescence. Data representative of n = 2 independent experiments **(b)** Germ-free animals were orally administered 80 μg MEV in PBS (GF + MEV), or PBS alone (GF + PBS), twice-weekly for 3 weeks. Whole liver was then collected and RNAseq performed and differences in individual transcriptome represented on a PCA plot (n = 5 per group). **(c)** Volcano plot illustrating differentially down- and up-regulated genes in GF + MEV vs. GF + PBS mice. **(d)** Enrichment plot for GSEA Reactome pathway analysis of liver RNA transcriptome data, with significant enrichment in pathway associated with ‘FOXO mediated transcription’ and ‘Gluconeogenesis’ in GF + MEV livers **(e)** Specific-pathogen free (SPF) mice were administered 1 × 10^10^ MEV (MEV), or PBS as control (PBS) daily for 5 weeks, and liver gene expression of *Pck1* and *G6pc* quantified by qPCR (n = 6 per group). RNAseq experiment representative of n = 1 experiment **(f)** Primary hepatocyte cell isolated from control C57BL/6 male mice was cultured for 18 h in the absence (PBS) or presence of 3 × 10^9^ MEV (MEV), and gene expression level of *Pck1* and *G6pc* quantified by qPCR (n = 5 PBS, n = 6 MEV). Data representative of n = 2 independent experiments **(g)** HEPG2 human hepatocyte cell line was cultured for 18 h in the absence (PBS) or presence of 3 × 10^9^ MEV (MEV), and gene expression level of *PCK1* and *G6PC* quantified by qPCR (n = 6 per condition). Data representative of n = 5 independent experiments **(h)** SPF mice were administered 1 × 10^10^ MEV (MEV), or PBS as control (PBS) daily for 5 weeks, and then pyruvate tolerance test performed, with blood glucose level measured to 2 h following intraperitoneal injection of pyruvate at a dose of 2.5 g/kg body weight following overnight fasting (n = 8 PBS and n = 6 MEV). Data representative of n = 1 experiment. All data are presented as mean ± SEM. ∗p < 0.05 and ∗∗p < 0.01 by Mann–Whitney test. For PTT experiment, overall difference was determined by two-way ANOVA.

To elucidate the potential role of MEV in host glucose homeostasis, we first determined whether MEV produced by gut bacteria can cross the intestinal barrier and translocate systemically. MEV was purified from cecal content and stool from control animals (Fig. S1a–b), fluorescently-tagged and orally administered to germ-free (GF) mice and translocation was tracked by *ex vivo* tissue imaging 12 h later. GF mice were used to eliminate potential competition from endogenous MEV produced by the gut microbiota for translocation. We found that MEV predominantly concentrated in the liver (Figure 1A), and to a lesser extent in the kidneys (Fig. S1c). To understand the impact of MEV on the liver, we administered MEV to GF mice orally twice weekly for 3 weeks and performed transcriptomic analysis on the liver. Principal component analysis (PCA) revealed clear separation of liver transcriptomes between MEV-treated and PBS-treated groups (Figure 1B). Genes related to gluconeogenesis including *Pck1*, *G6pc*, *Foxo1* and *Ppargc1a* were differentially upregulated in MEV-treated animals, compared to PBS-treated animals (Figure 1C). This was confirmed by pathway enrichment analysis, with the FOXO mediated transcription pathway significantly enriched (Figure 1D). FOXO1 is a key transcription factor that induces the expression of the key gluconeogenic enzymes PEPCK and G6P. Indeed, the gluconeogenesis pathway was also enriched in MEV-treated mice (Figure 1D), corresponding to higher *Pck1* and *G6pc* expression as validated by qPCR (Fig. S1d). Differentially expressed genes are provided (Supplemental Table 1). To validate this phenotype, we orally administered 1 × 10^10^ MEV to specific-pathogen free (SPF) mice daily for 5 weeks, and then quantified liver gene expression of *Pck1* and *G6pc*. In our hands, 50–100 mg of stool contains approximately 6.3 × 10^10^ to 1.26 × 10^11^ MEV, indicating that 1 × 10^10^ MEV is not an excessive dose and likely falls within the lower end of physiological ranges. Compared to PBS-treated animals, MEV-treated mice had increased liver expression of both *Pck1* and *G6pc* (Figure 1E). We further verified that MEV induced hepatic gluconeogenesis, upregulating the expression of genes encoding for PEPCK and G6P, under both *ex vivo* conditions with mouse primary hepatocytes (Figure 1F), and under *in vitro* conditions with the human hepatocyte cell line HEPG2 (Figure 1G). These results indicate that MEV act directly on hepatocytes to induce gluconeogenesis. We found that the effect of MEV on the expression of gluconeogenic genes in HEPG2 cells occurred at a threshold dose of approximately 3 × 10^9^ particles, with *G6PC* expression increasing at higher doses (Fig. S1e), with maximal effect at 10 × 10^9^ particles (data now shown). Finally, to determine the physiological significance of MEV on the host, SPF mice were administered PBS or MEV for 5 weeks, as described above. Pyruvate tolerance test was then performed, a gold standard method to assess the conversion of exogenous pyruvate to glucose *in vivo*, as a readout of gluconeogenesis. Indeed, MEV-treated mice had higher blood glucose levels following fasting and administration of the gluconeogenic substrate pyruvate (Figure 1H). Together, these results demonstrate that MEV exposure increases liver gluconeogenesis both *in vivo* and *in vitro*, and that MEV acted directly on hepatocytes to induce gluconeogenesis.

### 2.2 Gut microbiota-derived extracellular vesicles promote hepatic inflammation and metabolic syndrome

**Figure 2:**
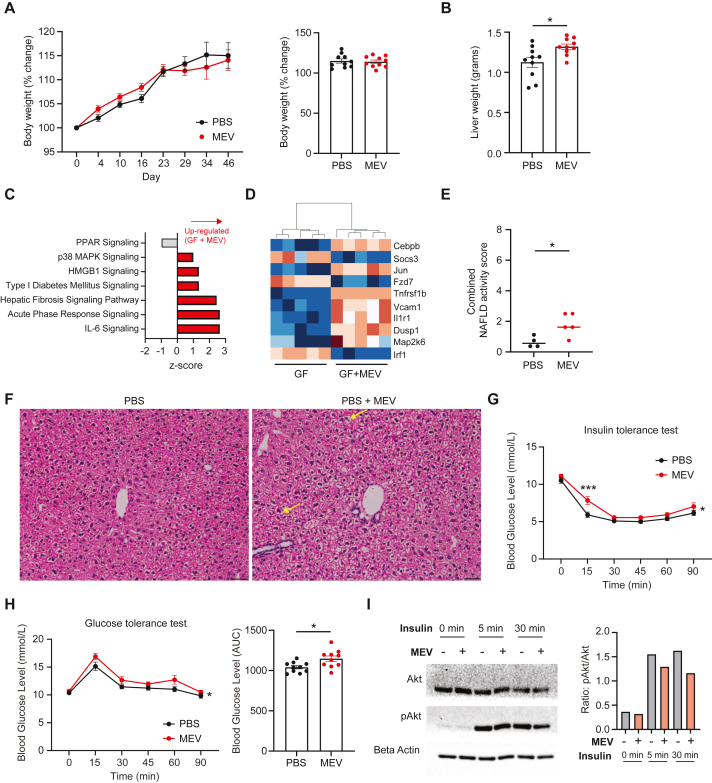
Gut microbiota-derived extracellular vesicles promote low-grade hepatic inflammation and insulin resistance. **(a)** SPF mice were administered 1 × 10^10^ MEV (MEV), or PBS as control (PBS) daily for 5 weeks. Graph represents % body weight change over the 5 weeks period (left) and scatter plot showing the percentage change at endpoint (right) (n = 10 mice per group). Data representative of n = 3 independent experiments. **(b)** Scatter plot showing whole liver weight following 5 weeks oral administration of MEV or PBS (n = 10 mice per group). Data representative of n = 2 independent experiments. **(c)** Germ-free animals were orally administered 80 μg MEV in PBS (GF + MEV), or PBS alone (GF + PBS), twice-weekly for 3 weeks. Whole liver was then collected and RNAseq performed, and Qiagen Ingenuity pathway analysis performed, with **(d)** heatmap with key genes involved in the pathways shown. (n = 5 per group). Data represent of n = 1 experiment. **(e)** SPF mice were administered 1 × 10^10^ MEV (MEV), or PBS as control (PBS) daily for 5 weeks. Representative liver hematoxylin and eosin staining. Yellow arrows indicate hepatocyte ballooning. Scale bar represents 50 μm and **(f)** combined NAFLD activity score of liver histology samples. Data representative of n = 1 experiment and scores represent average scores of n = 2 independent scoring. **(g)** SPF mice were administered 1 × 10^10^ MEV (MEV), or PBS as control (PBS) daily for 5 weeks, and then oral glucose tolerance test performed, with blood glucose level measured to 90min following oral administration of glucose at a dose of 2 g/kg body weight following 6 h of fasting (n = 10 mice per group). **(h)** SPF mice were administered 1 × 10^10^ MEV (MEV), or PBS as control (PBS) daily for 5 weeks, and then insulin tolerance test performed, with blood glucose level measured to 90 min following intraperitoneal administration of insulin at a dose of 0.5U/kg body weight following 4 h of fasting (n = 10 mice per group). Data represent of n = 1 experiment. **(i)** Primary hepatocyte cell isolated from control C57BL/6 male mice was cultured for 18 h in the absence (PBS) or presence of 3 × 10^9^ MEV (MEV), and then stimulated with 10 nM insulin for 0, 5 or 30min and protein levels of Akt and p-Akt quantified by Western blot (each condition pooled from n = 3 replicates of n = 1 mice). Data representative of n = 2 independent experiments. All data are presented as mean ± SEM. ∗p < 0.05 by Mann–Whitney test. For oGTT and ITT experiments, overall difference was determined by two-way ANOVA.

Next, we sought to determine how increased exposure to MEV affects host metabolic health. Treatment with MEV for 5 weeks did not alter food intake (Fig. S2a), nor affect body weight gain (Figure 2A). MEV treatment did not affect inguinal white adipose tissue (WATi) weight but increased epididymal white adipose tissue (WATe) weight (Fig. S2b). Notably, it significantly increased liver weight (Figure 2B), a change often associated with inflammation [18]. This was confirmed by Ingenuity pathway analysis of our transcriptomic data (Figure 2C), revealing enrichment of pro-inflammatory pathways relating to IL-6 and acute phase response signalling, as well as HMGB1 signalling pathways. Genes common to these pathways like *Jun*, *Cebpb* and *Tnfrsf1b* are generally considered pro-inflammatory and were significantly upregulated in MEV treated animals (Figure 2D). Histological analysis revealed mild signs of liver inflammation in MEV-treated animals (Figure 2E–F), particularly increased hepatocyte ballooning (Fig. S2c), while no fibrosis was detected (data not shown). This was not accompanied by evidence of elevated systemic inflammation, with no differences in circulating levels of IL-1β, TNF, IL-6, MCP-1, KC and IL-12 (Fig. S2d), suggesting that this effect was local. Excessive gluconeogenesis is a feature of obesity, and chronic low-grade inflammation is a feature of metabolic syndrome. To determine the physiological implication of increased hepatic gluconeogenesis and hepatic inflammatory state, we tested the impacts of MEV on glucose metabolism. We performed glucose tolerance and insulin tolerance test on mice treated with PBS or MEV for 5 weeks, which revealed that MEV administration induced mild but significant insulin resistance (Figure 2G) and glucose intolerance (Figure 2H). Indeed, mouse primary hepatocyte that were pre-treated overnight with MEV had decreased Akt phosphorylation following stimulation with insulin, highlighting that MEV can directly induce insulin resistance (Figure 2I). Altogether, these results demonstrate that increased MEV exposure can lead to a mild but significant disruption to metabolic health by promoting glucose intolerance and insulin resistance. This phenotype was not attributed to increased systemic inflammation.

### 2.3 Host feeding patterns control the diurnal production of MEV to regulate hepatic gluconeogenesis

**Figure 3:**
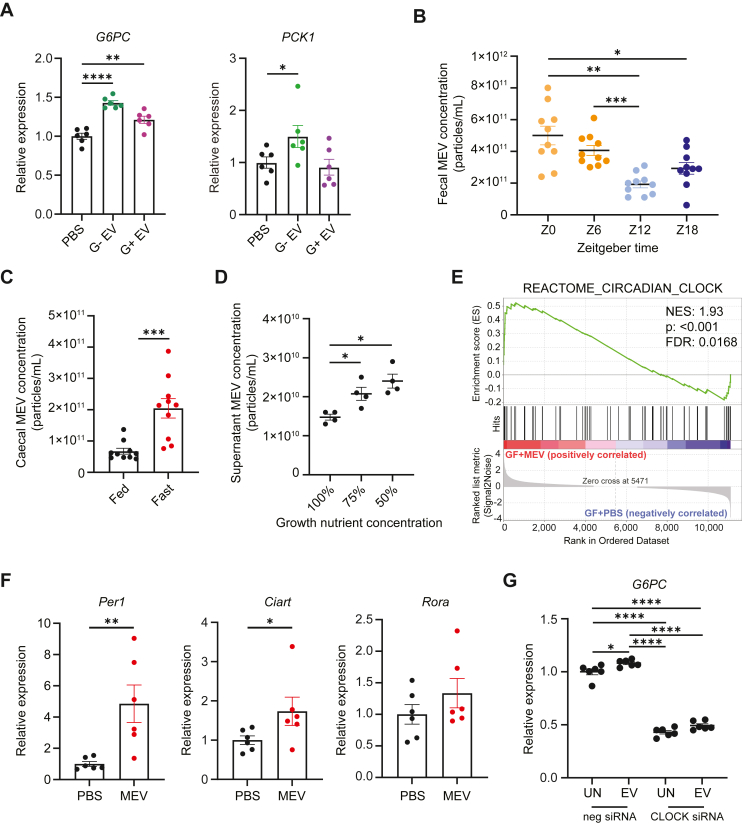
Gut microbiota-derived vesicles are produced rhythmically in response to host nutritional status and activate the circadian clock to promote gluconeogenesis. **(a)** HEPG2 hepatocyte cell line was cultured for 18 h in the absence (PBS) or presence of 3 × 10^9^ gram-negative EV (G- EV) from *Escherichia coli* or gram-positive EV (G+ EV) from *Enterococcus faecalis*, and gene expression level of *PCK1* and *G6PC* quantified by qPCR (n = 6 per condition). Data representative of n = 2 independent experiments. **(b)** Feces MEV concentration was quantified by Nanoparticle tracking analysis from the feces of control mice collected at Zeitgeber time 0 (start of light cycle), 6, 12 (start of dark cycle) and 18. Data represent of n = 1 experiment **(c)** Cecal MEV concentration was quantified by Nanoparticle tracking analysis from the feces of control mice that was fed ad libitum, or fasted overnight (18 h). Data representative of n = 1 experiment. **(d)***E. faecalis* was grown *in vitro* at decreasing concentration of nutrient and EV was isolated and quantified by Nanoparticle tracking analysis. Data representative of n = 2 independent experiments. **(e)** Germ-free animals were orally administered 80 μg MEV in PBS, or PBS alone, twice-weekly for 3 weeks. Whole liver was then collected and RNAseq performed, and enrichment plot for the GSEA Reactome ‘Circadian Clock’ pathway analysis shown. Data represent of n = 1 experiment. **(f)** SPF mice were administered 1 × 10^10^ MEV (MEV), or PBS as control (PBS) daily for 5 weeks, and then liver expression of *Per1*, *Ciart1* and *Rora* was quantified by qPCR. Data representative of n = 2 independent experiments. **(g)** HEPG2 hepatocyte cell line was reverse transfected with CLOCK-siRNA or control negative-siRNA and cultured for 18 h in the absence (PBS) or presence of 3 × 10^9^ MEV (MEV), and gene expression level of *PCK1* and *G6PC* quantified by qPCR (n = 6 per condition). Data representative of n = 3 independent experiments. All data are presented as mean ± SEM. ∗p < 0.05, ∗∗p < 0.01, ∗∗∗p < 0.001, ∗∗∗∗p < 0.0001 by Mann–Whitney test (c, f), ordinary one-way ANOVA followed by Tukey's multiple comparisons test (a, b, d) or two-way ANOVA followed by Tukey's multiple comparisons test (g).

MEV can promote hepatic gluconeogenesis via numerous pathways. We previously found that MEV modulated host immunity through the activation of TLRs, such as TLR4, via LPS present on the MEV surface [19]. LPS has also been shown to promote hepatic gluconeogenesis via the activation of the transcriptional regulator P300 [20,21]. We found that MEV did not induce hepatic gluconeogenesis through TLR signalling, with MEV still able to upregulate *PCK1* and *G6PC* expression in the presence of the NF-κB inhibitor BAY11-7082 (Fig. S3a), and the P300 inhibitor C646 (Fig. S3b), suggesting that mechanisms independent of LPS were involved. We further confirmed that MEV-associated LPS was not the mediator of hepatic gluconeogenesis, as EV derived from both *Escherichia coli* (gram-negative, enriched in LPS) and *Enterococcus faecalis* (gram-positive, lacking LPS) could induce *PCK1* and/or *G6PC* expression in HEPG2 cell line (Figure 3A). It was also not due to a general increase in proinflammatory signalling pathways, mediated by JNK (Fig. S3c). Other pathways known to induce gluconeogenesis, including ROS activation (Fig. S3d–f) and SIRT1 (Fig. S3g) were also not involved.

We hypothesized that MEV may possibly control hepatic gluconeogenesis by regulating the circadian clock, given the rhythmic expression of *PCK1* and *G6PC* observed in hepatocytes [2]. This hypothesis was supported by several observations. We found that the quantities of MEV produced in the gut peaked at similar Zeitgeber times that hepatic *Pck1* and *G6pc* expression are reported to be at the highest [22] (Figure 3B). Notably, MEV concentration was lowest during the start of the feeding phase (dark cycle, Zeitgeber time 12), while it was highest during the start of the fasting phase (light cycle, Zeitgeber time 0), when glucose demand is highest. This demonstrates that MEV are produced in a diurnal pattern, and this directly aligns with host glucose requirements. To further demonstrate the relationship between host nutritional status and MEV production, we subjected mice to starvation via overnight fasting. Starvation was linked to a dramatic increase in MEV concentrations in the gut, with approximately 3 times more MEV produced by the gut microbiota following overnight fasting compared to ad libitum fed animals (Figure 3C). In addition to starvation, we previously found that host dietary composition can affect MEV production, with a high-protein diet associated with higher gut MEV concentrations [19]. Indeed, we found that high protein content in diet was associated with higher hepatic expression of *Pck1* (Fig. S4a). Next, to directly test the relationship between nutrient status and bacterial EV production, we cultured *E. faecalis*, a common gut commensal microbe, at decreasing concentration of nutrients in growth media. We found that the quantities of EV produced was increased with decreasing nutrient concentration (Figure 3D), highlighting that nutrient depletion is associated with increased EV production. Interestingly, we found that gram-positive bacteria generally produced higher quantities of EV than gram-negative bacteria (Fig. S4b). To apply this to another physiological setting, we also found pregnant mice had increased MEV concentration during the third trimester of gestation (Fig. S4c), the period where both energy demands [23] and maternal gluconeogenesis [24] are reported to be at their highest. Additionally, our hepatic transcriptome data revealed enrichment in the circadian clock pathway in MEV treated animals (Figure 3E). The activation of circadian clock (via CLOCK/BMAL1) leads to the expression of clock-controlled genes, which includes the regulators period (PER) and cryptochrome (CRY) that negatively regulate CLOCK/BMAL1 activity [25]. It also induces the expression of other regulators such as *Rora* and *Ciart1*. Accordingly, we found that the expression of these genes was differentially upregulated in MEV-treated mice in our transcriptomic dataset (Supplemental Table 1). Consistent to this, SPF mice treated with MEV for 5 weeks also had upregulation of *Per1*, *Rora* and *Ciart1* compared to PBS-treated animals (Figure 3F), and this phenotype was also replicated in HEPG2 cell line treated with MEV *in vitro* (Fig. S4d). Interestingly, MEV administration did not induce gluconeogenesis in the kidneys (Fig. S4e), another major site of gluconeogenesis, suggesting that this effect is specific to the liver. This aligns with a previous report indicating that kidney gluconeogenesis is not regulated by circadian rhythm, unlike the liver [2]. We also did not see significant changes in the small intestine (Fig. S4f), with gluconeogenesis known to be triggered more strongly by nutrient availability at this site [26]. Finally, siRNA knockdown of CLOCK in HEPG2 cell line (Fig. S4g) lowered basal *G6PC* expression and abrogated MEV-induced upregulation of this gene (Figure 3G). We found that transfected cells had expression of *PCK1* at background levels (data not shown). Together, our data indicate that the MEV plays a role in supporting host glucose needs by enhancing hepatic gluconeogenesis, and this is associated with changes to the liver clock system.

## 3 Discussion

**Figure 4:**
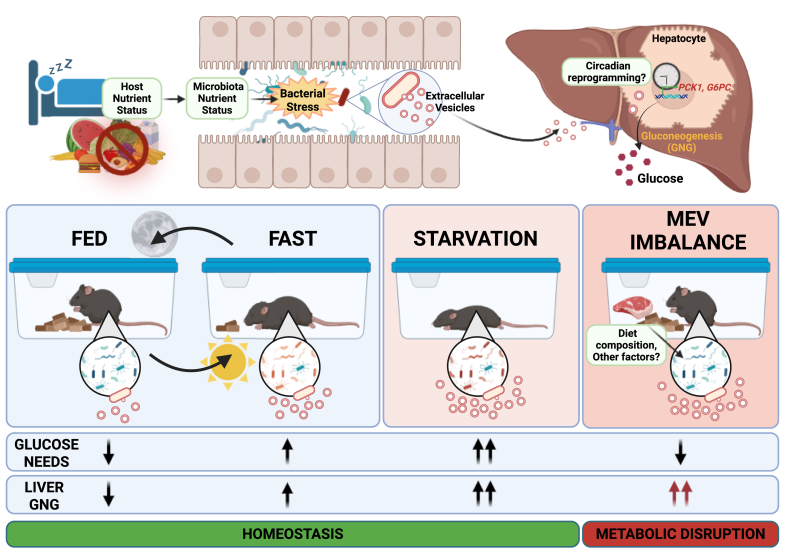
Model of gut microbiota-derived extracellular vesicles induction of host hepatic gluconeogenesis. Schematic of the interplay between host nutritional status, gut microbiota production of extracellular vesicles, and host hepatic gluconeogenesis. Host nutrient status mediates gut microbial stress, with fasting leading to bacterial stress and increased production of microbiota-derived extracellular vesicles (MEV). MEV can translocate to the liver, where it influence the hepatic circadian clock, and is associated with increased expression of *PCK1* and *G6PC*, and the upregulation of hepatic gluconeogenesis. Under normal conditions, the diurnal regulation of MEV associates with host glucose requirements, leading to homeostasis. However, the disruption of MEV production, such as improper diet or other factors, can stimulate gluconeogenesis despite the host being euglycemic, which may lead to metabolic disruption and predisposition to metabolic disorders. Created in BioRender. Tan, J. (2025) https://BioRender.com/qaenmir.

We show for the first time that the gut microbiota, via EVs, can activate hepatic gluconeogenesis. MEV production exhibit diurnal regulation, which is linked to host nutritional status, and appears to have evolved to support host glucose demands (Figure 4). MEV mediated this effect by acting as a potential peripheral Zeitgeber for the hepatic circadian system, and we hypothesize that this is a new pathway for host-gut microbe interactions. Indeed, greater quantities of MEV were found to be produced during periods of high glucose demands (i.e., fasting and pregnancy). This peripheral mode of regulation is applicable across mammalian species, regardless of whether they are nocturnal or diurnal, unlike central clock signalling which is regulated by the suprachiasmatic nucleus via light inputs. Our finding highlights several implications. Firstly, does different gut microbiota composition have different capacity for MEV production, and does this contribute in part to interindividual differences in baseline and fasting glucose levels? A minor increase in blood glucose level, along with other factors like genetics and dietary habits, might be sufficient to predispose to metabolic disorders. In our hands, we found that gram-positive bacteria generally produced higher levels of EVs than gram-negative bacteria, at least under *in vitro* conditions (Fig. S4b). This may explain the observation found in some studies that obese individuals have a higher Firmicutes:Bacteroidetes ratio [27]. Despite this, the fact that both gram-negative and gram-positive-derived EVs can activate gluconeogenesis suggests that the mechanism is not due to surface membrane components per se, but more likely driven by the delivery of intracellular cargo to hepatocytes. Host dietary composition can also affect MEV production. For example, we have previously shown that a high-protein diet was linked to greater quantities of gut MEV [19]. This may partly explain the established effects of a high-protein diet on host gluconeogenesis with a high-protein, carbohydrate-free diet linked to increased gluconeogenesis in humans [28]. We also validated this observation in mice utilizing 10 diets with different ratio of macronutrient composition, which revealed a positive association between liver *Pck1* expression and protein content in diet, with a minor interaction with carbohydrate content (Fig. S4a). As well as diet composition, irregular food intake common in modern societies may also impair the normal control of host glucose homeostasis by disrupting MEV production. While MEV act as a cue to support host glucose demands under normal conditions, it is possible that physiological disruption to MEV production (such as altered diet composition, irregular feeding and possibly medication etc.) may underlie increased metabolic disorders associated with a Western lifestyle.

Secondly, the fact that MEV directly activate gluconeogenesis *in vitro* indicates that MEV act independent of the diurnal actions of cortisol and glucagon on hepatic gluconeogenesis. This feature probably allows for the fine-tuning of hepatic gluconeogenesis, allowing the organism to adapt to different levels of hypoglycaemia. Indeed, the relative production in quantities of MEV was much greater in mice that were fasted overnight (Figure 3C) compared to the difference observed during normal rhythmic oscillation (Figure 3B). Aligned to this, we found that MEV acts on the expression of *PCK1* and *G6PC*, and in a dose-dependent fashion, at least with *G6PC in vitro* (Fig. S1e). The production of MEV was related to the nutritional status of the gut microbiota, directly linked to the host's nutritional intake. Stress (such as antibiotics) has been shown to promote bacterial EV production [29], and in this study, we provide evidence that nutrient availability is another factor regulating bacterial EV production.

In conclusion, our study provides new fundamental insights into the regulation of host glucose homeostasis. Assessing gut MEV profile holds significant clinical potential as a biomarker or risk factor for metabolic disorders, including gestational diabetes. Additionally, our study provides a mechanism for the control of host glucose homeostasis, via the alteration of MEV production. Although our short-term experiments revealed no overt systemic inflammation, chronically elevated MEV levels over the longer-term may trigger low grade inflammation and predispose to hepatic pathologies like non-alcoholic fatty liver disease and hepatocellular carcinoma, often associated with metabolic dysfunction. Inhibition of hepatic gluconeogenesis has been effective in ameliorating type 2 diabetes [30]. Thus, developing strategies aimed at reducing gut MEV production, i.e. by shaping dietary composition, have incredible potential in helping alleviate hyperglycaemia, possibly by tuning the liver clock system. This could form part of a strategy for the control or treatment of metabolic disorders like type 2 diabetes, by reducing hepatic gluconeogenesis.

## 4 Methods

### 4.1 Animals

Male C57BL/6 mice (6–8 weeks of age) were purchased commercially from Animal BioResources and housed at the Charles Perkins Centre animal facility. Animals were maintained under specific-pathogen-free conditions, with a standard 12 h light–dark cycle, at 25 °C and 50% humidity. Animals were fed ad libitum on AIN93G control diet, with free access to drinking water. Germ-free animals were purchased commercially from the Walter and Eliza Hall Institute of Medical Research and were housed at the Charles Perkins Centre germ-free animal facility. For biodistribution study, 80 μg of MEV was orally administered following a 2-hour fasting period. For other experiments, the administration of MEV were performed in the late afternoon, and faeces and cecal content were collected in the morning, unless otherwise stated. All animal experiments were performed under protocols approved by the University of Sydney Animal Ethics Committee (AEC).

### 4.2 Extracellular vesicle isolation

For the isolation of extracellular vesicles, cecum content or faecal pellets was homogenized in 0.02 μm filtered PBS and EV isolated by differential centrifugation, as described previously with slight modifications [19]. Briefly, the homogenate was centrifuged at 10,000×*g* for 20min at 4 °C followed by 18,000×*g* for 45min at 4 °C, before filtration through a 0.22 μm filter and centrifuged at 100,000×*g* for 2hr at 4 °C. EV pellet was resuspended in 0.02 μm filtered PBS. EV particle size and concentration were assessed by Nanoparticle Tracking Analysis using the Particle Metrix Zetaview, and analyzed with the Zetaview software (8.05.16 SP3) or the Nanosight NS300 (Malvern Instruments Limited), and analyzed with the NTA 3.2 software. MEV particle size distribution typically ranges from 133 to 425 nm (95% interval), with a median size of 211 nm. For fluorescent labelling of MEV, MEV was labelled with DiD dye at a final concentration of 5 μM for 30min at room temperature in the dark, quenched with ice-cold 0.02 μm filtered PBS, and then washed by ultracentrifugation as above.

### 4.3 RNA sequencing and data analysis

Total RNA was extracted from snap-frozen liver tissues using TRI Reagent (Sigma Aldrich) following the manufacturer's instructions, and RNA was purified using the RNeasy MinElute Cleanup Kit (Qiagen). Library preparation and Illumina HiSeq sequencing was done commercially (Azenta Life Sciences, previously Genewiz). Raw data had an average of 22,232,315 reads per sample with 94.02% of reads with quality of Q30 or above. Sequences were aligned to the GRCm38 mouse reference genome using STAR v2.7.3a with two-pass mapping [31]. Gene count was quantified using HTSeq and genes with less than 20 counts across all samples were filtered out prior to analysis. DESeq2 (1.42.0) was used for differential expression analysis [32] using R software (4.3.1). DESeq2 normalised counts and statistical outputs were used for downstream pathway analysis using Gene Set Enrichment Analysis [33] and Ingenuity Pathway Analysis (Qiagen). Raw and processed sequencing data are deposited in the NCBI GEO repository (GSE295797).

### 4.4 Quantitative PCR

Total RNA was extracted using TRI Reagent (Sigma Aldrich) following the manufacturer's instructions and cDNA generated using the High-Capacity cDNA Reverse Transcription Kit (ThermoFisher Scientific). qPCR was conducted using the Power SYBR™ Green PCR Master Mix (ThermoFisher Scientific) on a LightCycler® 480 Instrument II (Roche Life Science) and LightCycler® 480 v1.5.0 software. A list of primers used, and their sequences are provided in Supplemental Table 2.

### 4.5 Histological assessment

Liver tissues were fixed in 10% formalin and stored in 70% ethanol before processing and staining with H&E as previously described [34]. NAFLD Activity Score (Steatosis, Inflammation and Ballooning index) was used to score liver inflammation and damage, as previously described [35].

### 4.6 Cell culture

HEPG2 was cultured in DMEM supplemented with 10% FBS, 10 mM HEPES, 2 mM l-glutamine, 1 mM sodium pyruvate, 100units/ml penicillin and 100 μg/ml streptomycin. For experiments, HEPG2 cells were seeded into 6-well plate and grown until ∼80% confluence. For siRNA experiment, knockdown of CLOCK was performed using Silencer pre-designed siRNA (GGAACAAUAGACCCAAAGGtt and CCUUUGGGUCUAUUGUUCCtc) from Ambion (Life technologies) with Lipofectamine RNAiMAX (ThermoFisher) via reverse transfection, and experiments performed 48 h later. C57BL/6 mouse primary hepatocytes were isolated as previously described [36]. Briefly, liver was perfused with 25 ml of HBSS containing 5 mM calcium chloride and 0.05% collagenase IV maintained at 37 °C, and then hepatocytes teased out by gentle shaking on a petri dish. Hepatocytes were then centrifuged at 50×*g* for 30min at 22 °C, then resuspended in 37.5% Percoll solution and centrifuged for 500×*g* for 15min at 22 °C with minimal brake. RBC lysis was then performed, and hepatocytes resuspended in DMEM/F12 supplemented with 10% FBS, 5 mM HEPES, 2 mM l-glutamine, 2 mM sodium pyruvate, 1 μM dexamethasone, 100units/ml penicillin and 100 μg/ml streptomycin, and allowed to rest for 4 h on collagen coated plates (5 μg/cm^2^) before media removed and cell resuspended in fresh media without FBS and dexamethasone for experiments.

### 4.7 Metabolic assessments

For glucose tolerance test, mice were fasted for 6 h and were then given an oral bolus of glucose at a dose of 2 g/kg body weight. For insulin tolerance test, mice were fasted for 4 h, then 0.5 U/kg insulin was given by intraperitoneal injection. For pyruvate tolerance test, mice were fasted overnight, then given sodium pyruvate at a dose of 2.5 g/kg body weight. Blood glucose level was measured using a glucometer (AccuChek Advantage).

### 4.8 Bacterial cell culture

Frozen glycerol scrapping of *Enterococcus faecalis* (NCTC 775) was first inoculated in Brain Heart Infusion (BHI) broth and allowed to rest overnight, 37 °C shaking 200rpm. The culture was then diluted to OD0.1 in BHI broth and grown for 18 or 24 h as indicated. *Escherichia coli* (K-12 MG1655) was grown in Luria–Bertani (LB) broth instead. For some experiments, *E. faecalis* were grown in BHI broth diluted with salt solution (4.5 g/L NaCl and 2.5 g/L sodium phosphate dibasic).

### 4.9 Western blots

Samples were lysed by probe sonication (15s) in RIPA buffer supplemented with protease (Roche Mini Protease Inhibitor Cocktail) and phosphatase (Roche PhosSTOP) inhibitors before centrifuging (4 °C, 10min, 16,000×*g*). Protein concentration was quantified with bicinchoninic acid (BCA) assay (Sigma–Aldrich). Lysates were prepared for polyacrylamide gel electrophoresis under reduced conditions. Proteins were resolved by western blot using 4–12% Bis-Tris gels (BioRAD) and transferred to a nitrocellulose membrane (BioRAD). After blocking with 5% BSA in TBST, protein expression was detected using specific primary antibodies by incubating overnight at 4 °C (Cell Signalling Technology: phospho-AKT #4058; AKT #4685; β-actin #4970). All antibodies were detected using secondary rabbit IgG-horse radishperoxidase (Cell Signalling Technology #7074) and visualised by enhanced chemiluminescence using the Pierce ECL Western Blotting Substrate (ThermoFisher Scientific).

### 4.10 Statistical analysis

For comparison of two independent groups, the Mann–Whitney tests were used. For comparison of three or more groups, a one-way ANOVA with Tukey's multiple comparison test was used. For comparison of differences among group means over time, two-way ANOVA was used. For comparison of paired samples, non-parametric paired t-test was used. Mixture modelling was performed as previously reported [19,37]. P value < 0.05 were considered statistically significant with ∗p < 0.05; ∗∗p < 0.01; ∗∗∗p < 0.005; ∗∗∗∗p < 0.001.

## CRediT authorship contribution statement

**Jian Tan:** Writing – review & editing, Writing – original draft, Validation, Investigation, Data curation, Conceptualization. **Jemma Justine Taitz:** Writing – review & editing, Investigation, Data curation. **Duan Ni:** Writing – review & editing, Validation, Investigation, Data curation. **Camille Potier-Vilette:** Writing – review & editing, Validation, Investigation, Data curation. **Gabriela Pinget:** Writing – review & editing, Investigation. **Tamara Pulpitel:** Writing – review & editing, Investigation, Data curation. **Dragana Stanley:** Writing – review & editing, Investigation. **Ralph Nanan:** Writing – review & editing, Investigation. **Laurence Macia:** Writing – review & editing, Supervision, Funding acquisition, Conceptualization.

## Declaration of competing interest

The authors declare the following financial interests/personal relationships which may be considered as potential competing interests: Laurence Macia reports financial support was provided by Australian Research Council. Laurence Macia reports a relationship with Sanofi that includes: employment. L.M. is a current employee of the Translational Science Hub Global Sanofi Vaccines R&D Brisbane, Australia. Her contribution to this work was done when she was an employee of the University of Sydney. The other authors declare no competing interests. If there are other authors, they declare that they have no known competing financial interests or personal relationships that could have appeared to influence the work reported in this paper.

## Data Availability

Raw and processed sequencing data are deposited in the NCBI GEO repository (GSE295797).
