# Supplementary material for: Gut microbiota-derived extracellular vesicles exhibit diurnal regulation and activate hepatic gluconeogenesis
Source: Mol Metab. 2025 Jun 6;98:102180. doi: 10.1016/j.molmet.2025.102180 (PMC12221565; doi:10.1016/j.molmet.2025.102180)
Supplement: Multimedia component 2 [file mmc2.pdf]

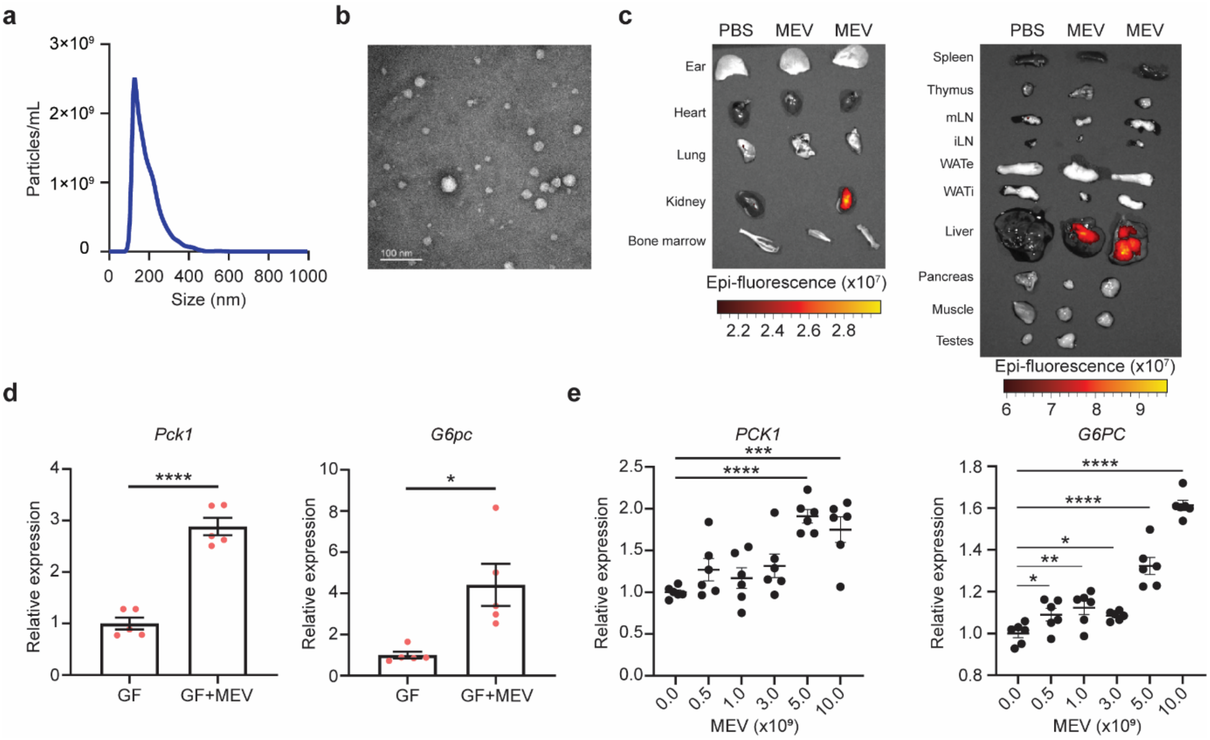

Figure S1: Gut microbiota-derived extracellular vesicles induce hepatic gluconeogenesis in vivo and in vitro. (a) Representative plot of nanoparticle tracking analysis of microbiota-derived extracellular vesicles (MEV) isolated from control mice and **(b)** representative transmission electron microscopy image of isolated MEV. **(c)** Germ-free animals were orally administered 80µg of DiD-labelled microbiota-derived extracellular vesicles (MEV) (n=2) or PBS as control (n=1). 12 hours later, skin (ear), heart, lung, kidney, bone marrow, spleen, thymus, mesenteric lymph nodes (mLN), inguinal lymph nodes (iLN), epididymal white adipose tissue (WATe), inguinal white adipose tissue (WATi), liver. Pancreas, muscle and testes were collected and fluorescence quantified ex vivo using the IVIS (In vivo imaging system). Relative fluorescence represented as Epi-fluorescence. Data represent of n=1 experiment. **(d)** Germ-free animals were orally administered 80µg MEV in PBS (GF+MEV), or PBS alone (GF), twice-weekly for 3 weeks. Whole liver was then collected, RNA extracted and gene expression of PEPCK and G6P quantified by qPCR. Data represent of n=1 experiment. **(e)** HEPG2 hepatocyte cell line was cultured for 18 hours in the presence of 0.0, 0.5, 1.0, 3.0, 5.0 or 10.0x10^9 MEV, and gene expression level of *PCK1* and *G6PC* quantified by qPCR (n=6 per condition). Data representative of n=2 independent experiments. All data are presented as mean±SEM. *$p<0.05$, **$p<0.01$, ***$p<0.001$ and ****$p<0.0001$ by Mann-Whitney test when compared to untreated (e).

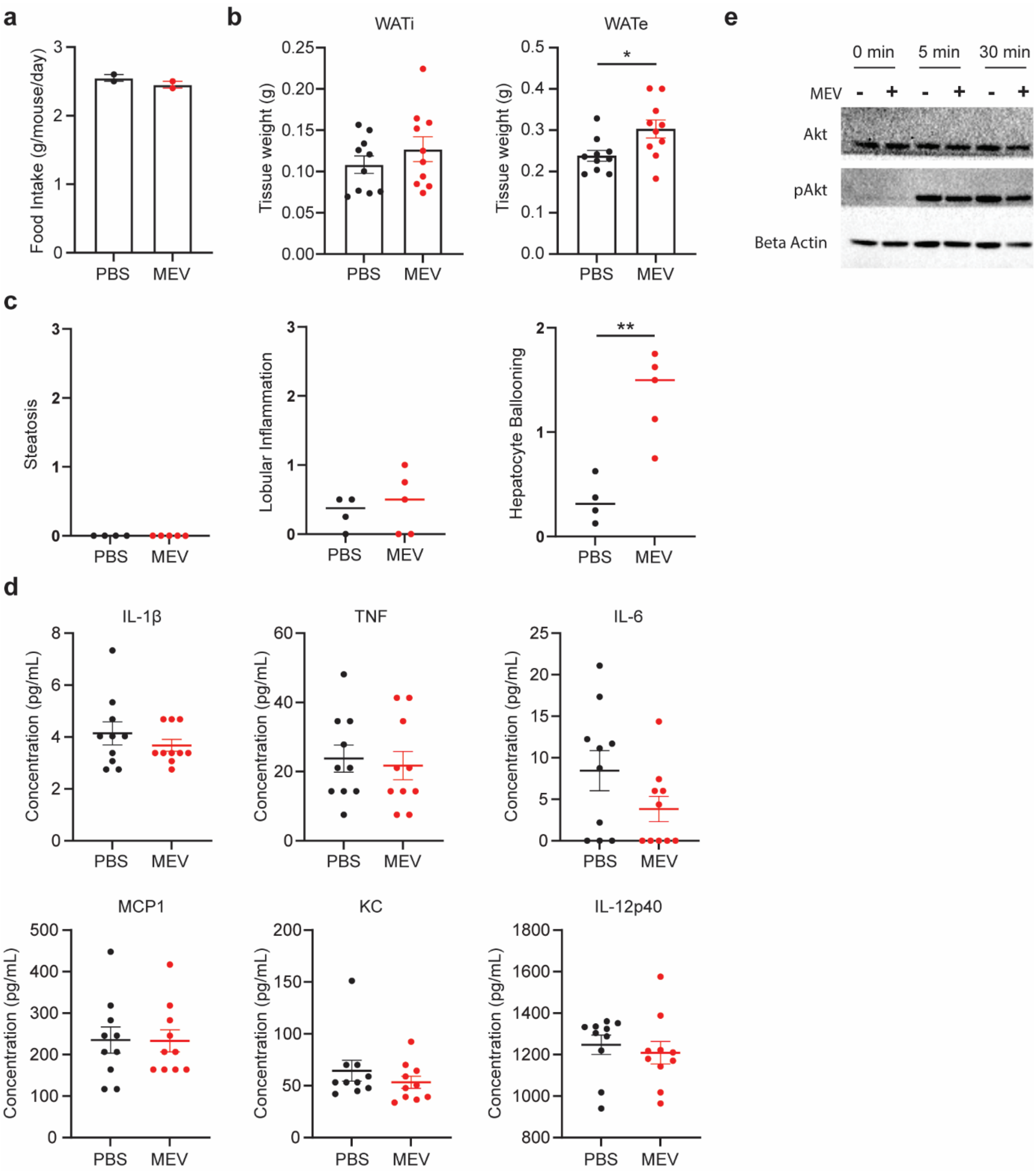

Figure S2: Gut microbiota-derived extracellular vesicles promote low-grade hepatic inflammation and insulin resistance. (a) SPF mice were administered 1x10^{10} MEV (MEV), or PBS as control (PBS) daily for 5 weeks. Graph represents average food intake (g/mouse/day). Each dot represents the average intake of one cage with n=5 animals per cage and **(b)** Scatter plot showing the weight of inguinal white adipose tissue (WATi) and epididymal white adipose tissue (WATe) weight. (n=10 mice per group). Data representative of n=2 independent experiments. **(c)** NAFLD activity scores comprising Steatosis, Lobular Inflammation and Hepatocyte ballooning scores. Data representative of n=1 experiment and scores represent average scores of n=2 independent scoring. **(d)** SPF mice were administered 1x10^{10} MEV (MEV), or PBS as control (PBS) daily for 5 weeks and plasma was collected for cytokine quantification using the Bio-Plex Pro Mouse Cytokine Multiplex Immunoassay. Data representative of n=2 independent experiments. All data are presented as mean±SEM. *$p < 0.05$ by Mann-Whitney test.

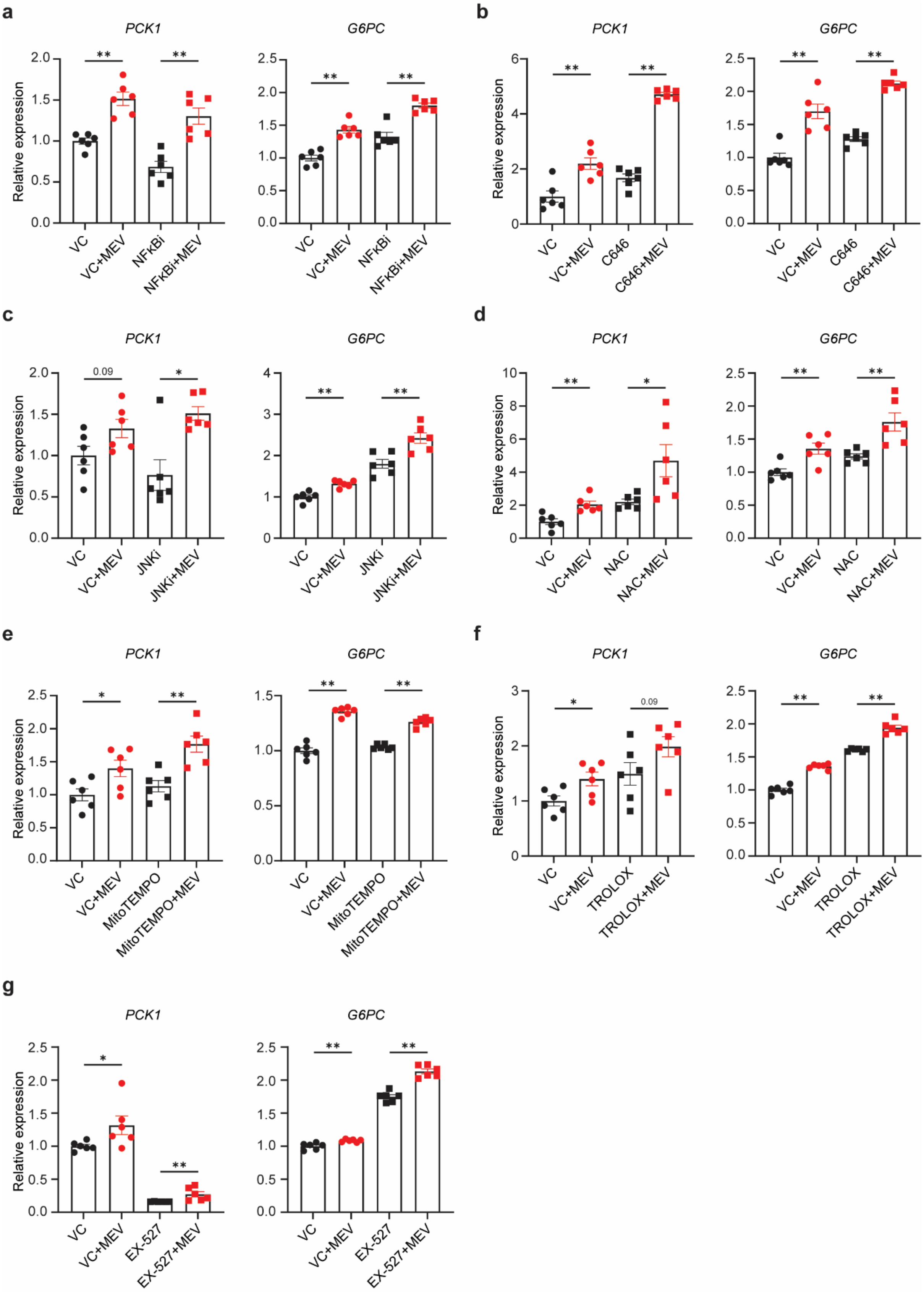

Figure S3: Gut microbiota-derived vesicles does not promote hepatic gluconeogenesis through activation of inflammatory and cellular-stress related pathways. HEPG2 hepatocyte cell line was cultured for 18 hours in the absence (vehicle control; VC) or in the presence of $3x10^9$ MEV (VC+MEV), in the absence or presence of **(a)** 10μM NFκB inhibitor BAY11-7082, **(b)** 20μM p300 inhibitor C646, **(c)**

20μM JNK inhibitor SP600125, the antioxidants **(d)** 10mM N-acetyl cysteine, **(e)** 10μM MitoTEMPO and **(f)** 10μM TROLOX, **(g)** 10μM SIRT1 inhibitor EX-527, and gene expression level of *PCK1* and *G6PC* quantified by qPCR (n=6 per condition). Cells were pre-treated with the inhibitor for at least 30min prior to the addition of MEV. Results represent n=2 independent experiments. All data are presented as mean±SEM. *$p < 0.05$ and **<0.01 by Mann-Whitney test between untreated and MEV-treated samples for each condition.

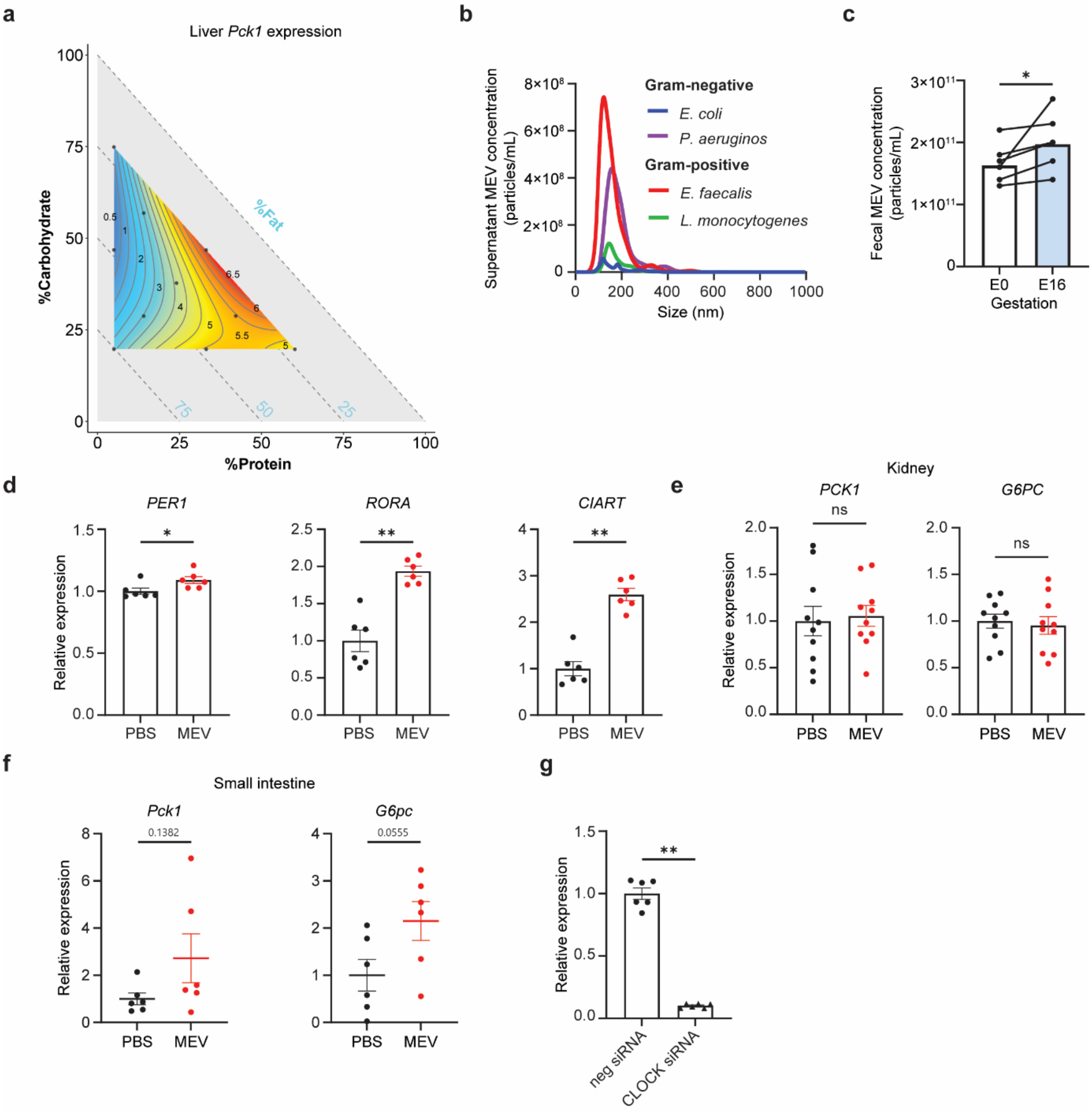

Figure S4: Gut microbiota-derived vesicles is produced rhythmically in response to host nutritional status and activates the circadian clock to promote gluconeogenesis.

(a) Contribution of macronutrient composition to liver *Pck1* expression (n = 7–8 per diet) was modelled by mixture model and represented on a right-angled mixture triangle comprising of carbohydrate (y axis), protein (x axis) and fat (hypotenuse) with liver *Pck1* expression (expression relative to the housekeeping gene *Rpl13a*, numbers on isolines) as the response variable. Red represents higher levels of *Pck1* expression while blue represents lower levels of *Pck1* expression in the nutrient mixture space. Each dot represents one of the 10 diets used for modelling response surface. Data representative of n=2 independent experiments. **(b)** Representative relative production of EV produced in culture *in vitro* by *Escherichia coli*, *Pseudomonas aeruginosa*, *Enterococcus faecalis* and *Listeria monocytogenes* following 18 hours of culture. Data representative of n=2 independent experiments. **(c)** MEV was quantified by Nanoparticle tracking analysis from feces collected from pregnant mice prior to gestation (E0) or at the third trimester of gestation (E16) (n=5 per group). Data represent of n=1 experiment. **(d)** HEPG2 hepatocyte cell line was cultured for 18 hours in the absence (PBS) or presence of 3×10^9 MEV (MEV), and gene expression level of *PER1*, *RORA* and *CIART* quantified by qPCR (n=6 per condition). Data representative of n=2 independent experiments. **(e-f)** Specific-pathogen free (SPF) mice were administered 1×10^{10} MEV (MEV), or PBS as control (PBS) daily for 5 weeks, *Pck1* and *G6pc*

gene expression quantified by qPCR in (e) kidney (n=10 per group) and (f) small intestine (n=6 per group). Data represent of n=1 experiment. **(g)** HEPG2 hepatocyte cell line was reverse transfected with CLOCK-siRNA or control negative-siRNA and cultured for 18 hours in the absence (PBS) and gene expression level of CLOCK quantified by qPCR (n=6 per condition). Data representative of n=3 independent experiments. All data are presented as mean±SEM. $^{*}p < 0.05$ and $^{**}<0.01$, by non-parametric paired t-test (c) or Mann-Whitney test (d-g).

Supplemental Table 2

Gene	Specie	Forward (5'-3')	Reverse (5'-3')
GAPDH	Human	GAAGGTGAAGGTCGGAGTCA	CAGAGTTAAAAGCAGCCCTGG
ACTB	Human	GACGACATGGAGAAAATCTG	ATGATCTGGGTCATCTTCTC
PCK1	Human	ATTCTGGGTATAACCAACCC	GTTGATGGCCCTTAAATGAC
G6PC	Human	ACTGTGCATACATGTTCATC	TGAATGTTTTGACCTAGTGC
CLOCK	Human	ACTACAAGACGAAAACGTAG	CATCTCTGTCAACAATCGAG
PER1	Human	ACACTTCAGAACCAGGATAC	AGTGGAACCATAGAAGACTC
CIART1	Human	AAGGATTTATACCTCCTCTCAC	TCTTTAACATCCCTTCTACCTG
RORA	Human	GCCATCAAAATTACAGAAGC	ATAAACACCACCTCTAGAGAAC
Rpl13a	Mouse	ATCCCTCCACCCTATGACAA	GCCCCAGGTAAGCAAACTT
Pck1	Mouse	AATATGACAACTGTTGGCTG	AATGCTTTCTCAAAGTCCTC
G6pc	Mouse	TTCAAGTGGATTCTGTTTGG	AGATAGCAAGAGTAGAAGTGAC
Clock	Mouse	AAGTGACTCATTAACCCCTG	CTATGTGTGCGTTGTATAGTTC
Per1	Mouse	GTTCTCATAGTTCCTCTTCTG	GTGAGTTTGTACTCTTGCTG
Ciart	Mouse	AGTCAAGAGATCAAGAGACG	GAAGCTACTTAATCCTCTGTC
Rora	Mouse	GAGTTTGTGTTCTATGCACC	CCTTGCATATTAGCTTGGTTAG
